# Acknowledgement to the reviewers of GMS Journal for Medical Education

**DOI:** 10.3205/zma001294

**Published:** 2020-02-17

**Authors:** Martin R. Fischer, Götz Fabry

**Affiliations:** 1 GMS Journal for Medical Education, Chief Editor, Erlangen, Germany; 2 University Hospital, LMU Munich, Institute for Medical Education, Munich, Germany; 3 Albert-Ludwig-Universität Freiburg, Medizinische Fakultät, Abteilung für Medizinische Psychologie und Soziologie, Freiburg, Germany

## Acknowledgement

We gratefully acknowledge the valuable contribution of the following peer reviewers, who supported our Journal in 2019. We are looking forward to continuing our collaboration!

## Reviewers in alphabetical order

- Kristina Ackel-Eisnach, Landau
- Simone Alvarez, Heidelberg
- Timo Astfalk, Rostock
- Cadja Bachmann, Rostock
- Joy Backhaus, Würzburg
- Daniel Bauer, Bern (CH)
- Marianne Behrends, Hannover
- Katrin Bekes, Wien (A)
- Martin Bergold, Oldenburg
- Markus Berndt, München
- Anja Böckers, Ulm
- Martin Boeker, Freiburg i. Br.
- Klaus Böhme, Bochum
- Raphael Bonvin, Fribourg (CH)
- Erich Brenner, Innsbruck (A)
- Irene Brunk, Berlin
- Till Bugaj, Heidelberg
- Andreas Burger, Bochum
- Jean-Francois Chenot, Greifswald
- Bas De Leng, Münster
- Ulrich Decking, Düsseldorf
- Nicole Deis, Mainz
- Clémence Delmas, Bern (CH)
- Kim Deutsch, Mainz
- Sabine Drossard, München
- Jan P.Ehlers, Witten
- Simone Ehrhard, Bern (CH)
- Maren Ehrhardt, Hamburg
- Mona Eulitz, Witten
- Götz Fabry, Freiburg
- Maximilian Fink, München
- Volkhard Fischer, Hannover
- Johannes Forster, Merzhausen
- Peter Frey, Bern (CH)
- Angelika Hiroko Fritz, Essen
- Robert Gaschler, Hagen
- Rainer Gaupp, Basel (CH)
- Karim Gawad, Bielefeld
- Kirsten Gehlhar, Oldenburg
- Waltraud Georg, Zürich (CH)
- Trevor John Gibbs, Dundee (UK)
- Marianne Giesler, Freiburg
- Maryna Gornostayeva, Mainz
- Martin Gössl, Graz (A)
- Jan Griewatz, Tübingen
- Lorenz Grigull, Hannover
- Christian Gruber, Hannover
- Markus Gulich, Ulm
- Sissel Guttormsen, Bern (CH)
- Stefan Gysin, Zürich (CH)
- Martin Haag, Heilbronn
- Petra Hahn, Freiburg
- Wolfgang Hampe, Hamburg
- Sigrid Harendza, Hamburg
- Roman Hari, Bern (CH)
- Christopher Harrison, Staffordshire (UK)
- Cordula Harter, Heidelberg
- Anja Härtl, Ausburg
- Wolf Hautz, Bern (CH)
- Sylvia Heenemann, Maastricht (NL)
- Inga Hege, Augsburg
- Susanne Heim, Göttingen
- Gunther Hempel, Leipzig
- Linn Hempel, Düsseldorf
- Eva Hennel, Bern (CH)
- Doreen Herinek, Berlin
- Wolfram J. Herrmann, Münster
- Stefan Herzig, Köln
- Sybille Hildenbrand, Tübingen
- Tanja Hitzblech, Berlin
- Matthias Hofer, Düsseldorf
- Anke Hollinderbäumer, Mainz
- Henrike Hölzer, Berlin
- Angelika Homberg, Heidelberg
- Bert Huenges, Bochum
- Sören Huwendiek, Bern (CH)
- Paul Jansen, Kamen-Heeren
- Stefanie Joos, Tübingen
- Stephanie Jungmann, Mainz
- Sylvia Kaap-Fröhlich, Zürich (CH)
- Juliane Kämmer, Berlin
- Niklas Keller, Berlin
- Matthew Kerry, Zürich (CH)
- Janna-Lina Kerth, Düsseldorf
- Jan Kiesewetter, München
- Christin Kleinsorgen, Hannover
- Thomas Kloppe, Hamburg
- Michael Knipper, Giessen
- Ursula Kriesen, Rostock
- Klaus-Dietrich Kröncke, Düsseldorf
- Susanne Kühl, Ulm
- Thomas Kühlein, Erlangen
- Sebastian Kuhn, Mainz
- Sandy Kujumdshiev, Leipzig
- Katharina Langer-Fischer, Ulm
- Wolf Langewitz, Basel (CH)
- Ronny Lehmann, Heidelberg
- Christin Löffler, Rostock
- Mark Lüdde, Kiel
- Sabine Ludwig, Berlin
- Cornelia Mahler, Tübingen
- Jan Matthes, Köln
- Juliane Meng-Hentschel, Bern (CH)
- Anika Mitzkat, Heidelberg
- Parisa Moll-Khosrawi, Hamburg
- Andreas Möltner, Heidelberg
- Sören Moritz, Köln
- Katja Anne Moschberger, Berlin
- Brigitte Müller-Hilke, Rostock
- Ulrike Nachtschatt, Innsbruck (A)
- Gerald Neitzke, Hannover
- Jan Nijhuis, Maastricht (NL)
- Zineb Miriam Nouns, Berlin
- Dino Carl Novak, Berlin
- Falk R. Ochsendorf, Frankfurt/Main
- Dorothea Penders, Berlin
- Harm Peters, Berlin
- Swetlana Philipp, Jena
- Gominda G. Ponnamperuma, Nugegoda (LKA)
- Ingrid Preusche, Wien (A)
- Wolfgang Prodinger, Innsbruck (A)
- Ann-Kathrin Rahm, Heidelberg
- Sabine Ramspott, Grafrath
- Eva Rasky, Graz (A)
- Florian Recker, Bonn
- Gilbert Reibnegger, Graz (A)
- Kathrin Reichel, Berlin
- Matthias Richter, Halle
- Laura Aurica Ritter, Giessen
- Katrin Rockenbauch, Leipzig
- Caroline Rometsch-Ogioun El Sount, Tübingen
- Marco Roos, Erlangen
- Thomas Rotthoff, Augsburg
- Caroline Ruiner, Trier
- Stefan Rüttermann, Frankfurt/Main
- Thomas C. Sauter, Bern (CH)
- Thorsten Schäfer, Bochum
- Stefan Schauber, Oslo (NO)
- Claudia Schlegel, Bern (CH)
- Ralf Schmidmaier, München
- Anita Schmidt, Erlangen
- Guidom Schmiemann, Bremen
- Kai Schnabel, Bern (CH)
- Eva Schönefeld, Münster
- Ina Schüler, Jena
- Susan Selch, Hamburg
- Thomas Shiozawa-Bayer, Tübingen
- Heidi Siller, Innsbruck (A)
- Anne Simmenroth, Würzburg
- Melanie Simon, Aachen
- Angelika Simonsohn, München
- Andreas Söhnel, Greifswald
- Marc Sohrmann, Lausanne (CH)
- Andreas Sönnichsen, Wien (A)
- Beat Sottas, Bourguillon (CH)
- Sandra Steffens, Hannover
- Sandra Steinböck, Wien (A)
- Verena Steiner-Hofbauer, Wien (A)
- Tina Stibane, Marburg
- Sylvère Störmann, München
- Christoph Stosch, Köln
- Ulrich Stößel, Freiburg
- Sylvia Stracke, Greifswald
- Fabian Stroben, Berlin
- Nils Thiessen, Kalkara (MLT)
- Christian Thrien, Köln
- Daniel Tolks, München
- Gert Ulrich, Zürich (CH)
- Maria Paula Valk-Draad, Witten
- Petra Verdonk, Amsterdam (NL)
- Jörg Vianden, La Crosse (USA)
- Barbara Vogel, Berlin
- Michaela Wagner-Menghin, Wien (A)
- Ursula Walkenhorst, Osnabrück
- Tobias Weberschock, Frankfurt/Main
- Birgit Wershofen, München
- Bärbel Wesselborg, Düsseldorf
- Simone Weyers, Düsseldorf
- Sabine Wiegmann, Berlin
- Mario Wijnen-Meijer, München
- Andreas Winkelmann, Neuruppin
- Ulrich Woermann, Bern (CH)
- Björn Zante, Bern (CH)
- Michaela Zupanic, Witten
- Tycho Zuzak, Herdecke

